# Agreement between a photogrammetry-derived five-compartment body-composition model and a three-compartment criterion among adult athletes

**DOI:** 10.1080/15502783.2025.2550177

**Published:** 2025-08-26

**Authors:** Kworweinski Lafontant, Claudia Gonzalez, Michelle Da Silva Barbera, Susan Kampiyil, Linh Nguyen, Sofea Smith, Jack Livingston, Jeffrey R. Stout, David H. Fukuda

**Affiliations:** University of Central Florida, Physiology of Work and Exercise Response Lab, Institute of Exercise Physiology and Rehabilitation Science, Orlando, FL, USA

**Keywords:** Body composition, Athletes, Accessibility, Model

## Abstract

**Background:**

Four- and five-compartment models are the widely accepted criterion for body composition, accounting for body mass, bone mineral content, total body water (TBW), and total body volume (TBV). However, the need for underwater weighing or air displacement plethysmography (ADP) to assess TBV has limited the practicality of multi-compartment models among athletes and practitioners. Recent advances in photo-based body composition assessments have allowed for quick and accessible assessments of TBV, and Bennett et al. recently created a 5C model based on photo-based TBV and BIA estimates. Yet, research comparing Bennett’s 5C model to a 3C criterion among athletes is scarce.

**Methods:**

We assessed body composition in 52 high intensity functional training athletes (men = 34, women = 18, age = 34.9 ± 11.2 years, BMI = 26.0 ± 3.5 kg/m^2^) from the greater Orlando, FL, region. All measurements were taken in the morning (7:30–11am) while participants were well hydrated (urine-specific gravity < 1.030), fasted for at least 3 h, free from caffeine for at least 12 h, and abstinent from strenuous physical activity and alcohol for at least 24 h. Wearing minimal and compressive clothing, all participants completed a multi-frequency bioelectrical impedance analysis (BIA; InBody 970®), ADP (BodPod®), and photogrammetry (Fit3D®) body composition assessment, in that order. Body mass, TBW, and BMC were extracted from the InBody 970®, while volume was extracted from both the BodPod® and the Fit3D. We applied Siri’s 3C model using BodPod®’s TBV and Bennett’s 5C model using the Fit3D’s TBV, with agreement being assessed using a Bland–Altman analysis 95% confidence intervals (CI) and limits of agreement (LoA). All statistical analyses were conducted using the “blandr” package within jamovi, and the threshold for statistical significance was set at *p* < 0.05.

**Results:**

Body volume from the BodPod (74.4 ± 15.1 L) significantly differed from the Fit3D®’s estimate (72.6 ± 14.7 L; bias = 1.81 L; 95% CI = 1.28, 2.34 L; LoA = −1.91,5.53 L *p* < 0.001). However, body fat percentage from the Siri 3C model (19.3 ± 6.4%) had excellent agreement with the Bennett 5C model (19.3 ± 6.6%; bias = 0.0008%; 95% CI = −0.002, 0.004%; LoA = −0.021,0.022%, *p* = 0.61).

**Conclusions:**

The Bennet 5C model appears to account for differences in photo-based TBV measures compared to ADP, providing excellent agreement in body composition assessments compared to a criterion 3C model. Using BIA and a Fit3D®, athletes may be able to obtain similar body composition assessments as 3C model with BIA and ADP, enabling greater accessibility of multi-compartment models. However, further research is needed as we were unable to compare Bennett’s 5C to a similar 5C criterion with a larger sample.

